# Lymphome T/NK nasal: à propos de deux cas rares

**DOI:** 10.11604/pamj.2018.30.141.7721

**Published:** 2018-06-19

**Authors:** Najoua Bouayad, Naoual Oubelkacem, Wafaa Bono, Ouafae Masbah, Touria Bouhafa, Abderrahman Elmazghi, Hassouni Khalid

**Affiliations:** 1 Service de Radiothérapie, CHU Hassan II-Fès, Maroc; 2 Service de Médecine Interne, CHU Hassan II-Fès, Maroc

**Keywords:** Nasosinusal, lymphome T/NK, immunohistochimie, radiothérapie, Nasosinusal, NK/T-cell lymphoma, immunohistochemistry, radiotherapy

## Abstract

Le lymphome T/NK est une affection grave et rare en Afrique et en Europe. Le traitement repose sur la radiothérapie et la chimiothérapie. Les auteurs rapportent deux observations de patients âgés respectivement de 55 et 52 ans, qui présentent une obstruction nasale bilatérale avec une rhinorrhée purulente, chez eux l'examen clinique a mis en évidence la présence d'une fente palatine. Le diagnostic n'a pu être posé qu'après une étude immuno-histochimique des biopsies. Les 2 patients ont bénéficié d'une chimiothérapie type CHOEP suivi d'une cure de radiothérapie. Le lymphome T/NK nasal est une forme agressive de lymphome non hodgkinien comportant des caractéristiques clinicopathologiques qui lui sont spécifiques. L'association de la chimiothérapie pour les stades avancés ne semble pas améliorer la survie par rapport à la radiothérapie seule qui reste le traitement de choix surtout pour les stades localisés. La localisation nasal du lymphome T /NK est rare. Le diagnostic repose sur l'étude immunohistochimique. Le traitement comprend chimiothérapie et radiothérapie. Ce type lymphome est de mauvais pronostic globalement, y compris sous traitement.

## Abstract (en)

NK/T-cell lymphoma is a severe and rare disorder in Africa and in Europe. Treatment is based on radiotherapy and chemotherapy. We here report two cases of elderly patients aged 55 years and 52 years, respectively, with bilateral nasal obstruction associated with purulent rhinorrhea. Clinical examination showed cleft palate. Diagnosis was made on the basis of immunohistochemical examination of biopsies. Both patients underwent CHOEP chemotherapy followed by radiotherapy. Nasal NK/T-cell lymphoma is an aggressive type of non-Hodgkin’s lymphoma with specific clinicopathologic features. Combining chemotherapy with radiotherapy in patients with advanced stage of the disease does not seem to improve survival compared with radiotherapy alone, which is the treatment of choice especially for localized stages. Nasal NK/T-cell lymphoma is rare. Diagnosis is based on immunohistochemical examination. Treatment includes chemotherapy and radiotherapy. This lymphoma has a poor overall prognosis, even with appropriate therapy.

## Introduction

Le lymphome NK/T est un type rare de lymphome non hodgkinien [1,2]. Il correspond à une entité clinique réalisant une nécrose qui débute préférentiellement dans les cavités nasales et le nasopharynx pour s'étendre aux structures osseuses centrofaciale dont l'évolution spontanée reste fatale. Son diagnostic anatomopathologique n'est pas toujours évident. Les outils modernes d'immunophénotypage et de biologie moléculaire ont permis d'isoler ce type de lymphome. Le traitement repose sur la radiothérapie et la chimiothérapie. La localisation nasosinusienne des lymphomes T/NK est rare. Nous en rapportons deux cas et discutons la particularité de cette localisation, la difficulté diagnostique et les modalités thérapeutiques.

## Patient et observation

### Cas 1

**Figure 1:**
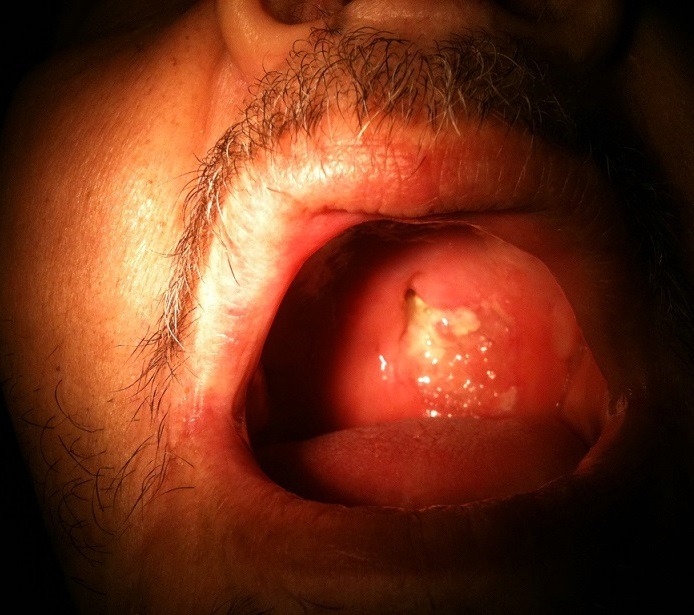
Ulcération nécrotique de la muqueuse du palais dur chez un patient de sexe masculin de 55 ans

**Figure 2:**
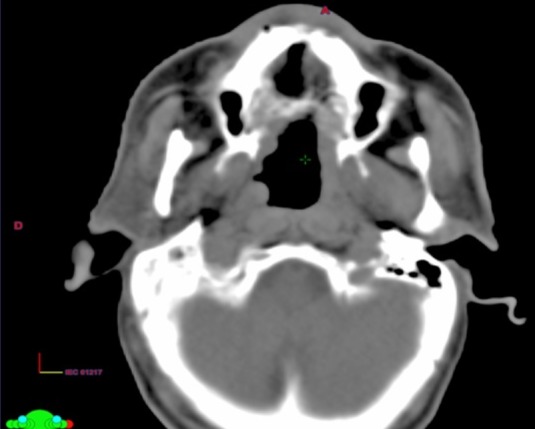
Coupe TDM axiale: processus tissulaire homogène étendu à la fosse nasale

Un homme de 55 ans, consultait pour une obstruction nasale bilatérale d'installation progressive, associée à de rhinorrhée purulente résistante au traitement habituel évoluant depuis 8 mois dans un contexte d'apyrexie et d'altération de l'état général. L'examen oto-rhino-laryngologique objectivait une formation bourgeonnante, saignant au contact, comblant toute la fosse nasale droite. A noter que l'examen endoscopique était impossible vu l'importance de la masse. L'examen de la cavité buccale montrait une ulcération du palais dur (Figure 1). Le reste de l'examen clinique était normal notamment celui des aires ganglionnaires. La tomodensitométrie nasosinusienne objectivait un processus tumoral nasal droit envahissant le sinus maxillaire homolatéral, accompagnée d'une ostéolyse du bord alvéolaire du maxillaire supérieur et du palais dur (Figure 2). L'étude histopathologique des prélèvements biopsiques de la cavité nasale droite et du palais osseux réalisés sous anesthésie locale montrait un processus diffus non différencié à cellules rondes larges, l'immunohistochimie a fait le diagnostic de certitude de lymphome nasal T/NK; les éléments lymphoïdes étaient positifs pour le CD3, CD2 et CD56. Le bilan d'extension était négatif. Le lymphome était classé stade IE selon la classification d'Ann Arbor (Tableau 1). Le patient a bénéficié d'une polychimiotherapie (4 cures selon le protocole CHOEP: adriamycine, vincristine, cyclophosphamide, étoposide et prédnisone) suivie d'une radiothérapie externe loco-régionale à la dose de 50Gy en 25 séances (2 Gy/fr; 5 fr/sem). Le malade a bénéficié d'une reconstruction de la fente palatine après 1 an de la fin d'irradiation. Il est en rémission complète clinique et radiologique après un recul de 25 mois.

**Tableau 1:** Classification d’Ann Arbor

STADE I	atteinte d'une seule aire ganglionnaire ou d'une seule structure lymphoïde: rate, thymus, anneau de Waldeyer (qui correspond aux différentes amygdales) ou d'une seule localisation d'un territoire extra-ganglionnaire contigu.
STADE II	atteinte de deux aires ganglionnaires, ou davantage, du même côté du diaphragme, pouvant être associée à une atteinte extra-ganglionnaire de continuité.
STADE III	atteinte ganglionnaire située de part et d'autre du diaphragme, pouvant être accompagnée éventuellement d'une atteinte splénique (rate) (III S)
STADE IV	atteinte d'une ou plusieurs localisations extra-ganglionnaires avec ou sans atteinte ganglionnaire.
STADE A/B	absence (A) ou présence (B) d'au moins un signe général
	- fièvre, au moins 38° pendant 8 jours consécutifs sans infection patente,
	- sueurs nocturnes,
	- perte de poids inexpliquée d'au moins 10% du poids du corps au cours des 6 mois précédents.
STADE E	envahissement d'une structure extra-lymphatique correspondant
	- soit à la seule atteinte de la maladie (I E)
	- soit à une extension de contiguïté d'une atteinte ganglionnaire (II E ou III E).
STADE X	c'est la présence d'une masse tumorale importante (ganglion de plus de 10 cm de diamètre ou rapport médiastino-thoracique supérieur à un tiers

### Cas 2

**Figure 3:**
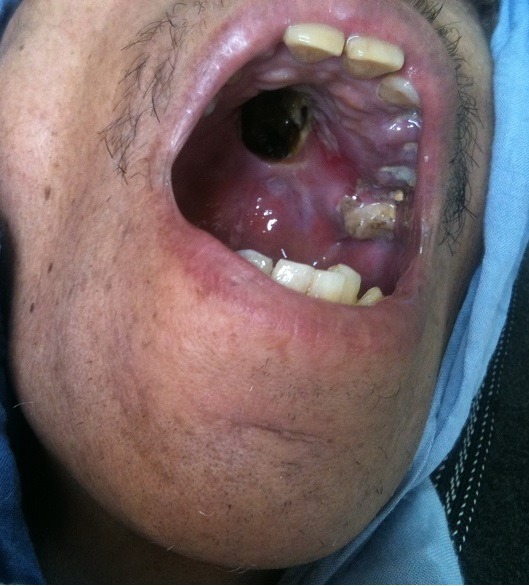
Ulcération du palais dur chez un patient de 52 ans

Un patient de 52 ans présente depuis 1 an une ulcération douloureuse de la muqueuse palatine en regard du palais dur. Elle grossissait progressivement jusqu'à éroder l'os du palais dur. A l'examen clinique, une perte de substance muqueuse et osseuse du palais dur, les berges étaient inflammatoires (Figure 3). Le reste de l'examen somatique était normal, notamment sans adénopathies locorégionale. Au scanner cervico-facial, une lyse de la cloison nasale et des cloisons nasosinusiennes secondaire à la présence d'un processus lésionnel tissulaire des fosses nasales mesurant 70 mm de diamètre antéropostérieur, étendu aux orifices choannaux et aux cellules ethmoïdales antérieures. L'étude immuno-histochimique de la biopsie qui a été réalisé sous anesthésie générale, a permis de trancher le diagnostic d'un lymphome T/NK. Les éléments lymphoïdes étaient positifs pour le CD45, négatifs pour le pancyto-kératine, positifs pour CD56, CD3 et négatifs pour CD20. Le bilan d'extension comportant une TDM thoraco-abdomino-pelvienne et une biopsie ostéo-médullaire était négatif. Le patient a été traité par une polychimiothérapie, 4 cures selon protocole de CHOEP, suivie d'une radiothérapie externe à la dose de 50 Gy en 25 fractions. Malade en bon contrôle clinique après un recul de 14 mois.

## Discussion

Anciennement dénommé « granulome malin centrofacial ». L'Organisation mondiale de la santé (OMS) a renommé dans sa classification cette entité par le terme extranodal natural killer (NK) T-cell lymphoma nasal type. Le lymphome T/NK est moins fréquent en Europe et en Amérique du Nord qu'en Asie et en Amérique du Sud [3]. Il touche plutôt l'adulte jeune de sexe masculin ce qui correspond à nos deux observations. En effet, le sex-ratio Homme/femme est de 3/1 et l'âge médian au moment du diagnostic est de 50-60 ans [4,5]. L'âge moyen de nos patients était de 53,5 ans. Le lymphome T/NK est de pathogénie inconnue. La présentation clinique est dominée par les signes locaux tels qu'une obstruction nasale unilatérale, rhinorrée purulente et/ou striée de sang, une épistaxis récidivante et un tableau de sinusite chronique [4,6]. Les lésions ulcéro nécrotiques peuvent siéger au niveau des fosses nasales et des sinus (70%), de l'anneau de Waldeyer (38%), de la cavité buccale (14%), du larynx et de l'hypopharynx (10%) [4,5]. Le lymphome T/NK est une affection rare caractérisée par son polymorphisme clinique pouvant expliquer les problèmes diagnostiques et le retard de prise en charge thérapeutique souvent observé. En effet, le mode de révélation de cette pathologie est peu spécifique pouvant même dérouter le clinicien. Le diagnostic repose sur l'étude immunohistochimique des biopsies. En effet, l'immunohistochimie permet de confirmer l'origine NK, cela en révélant une expression du CD2, du CD56 et des marqueurs de cytotoxicité (T1a, Granzyme B, perforine). Le CD5, le CD4 et le CD8 sont négatifs. Le CD3 est inconstamment exprimé (absence en surface du marqueur CD3) [7]. L'association de ces lymphomes T/NK avec le virus EBV est quasi-constante. Dans notre cas, il a fallu cette étude immuno-histochimique pour trancher. Le problème de diagnostic différentiel se pose avec d'autres lésions telles que la granulomatose de Wegener [8] la syphilis, la tuberculose, les autres tumeurs malignes et la consommation de cocaïne. La TDM permet d'orienter le diagnostic, elle retrouve souvent un aspect en faveur d'une tumeur solide, peu ou non rehaussée par l'injection de produit de contraste. La destruction des structures osseuses est observée dans moins de la moitié des cas, surtout dans les tumeurs volumineuses. L'IRM a un intérêt dans le bilan d'extension aux structures adjacentes et permet de différencier le processus tumoral d'un aspect inflammatoire. La biologie retrouve constamment un syndrome inflammatoire. Cependant, le lymphome T/NK est fortement associé à l'infection par l'EBV surtout en Asie [4]. Cette infection est associé à un mauvais pronostic avec une fréquence des rechutes locales, une possibilité d'extension vers d'autres sites extra ganglionnaires et de l'apparition d'un syndrome d'activation macrophagique [9]. C'est la complication la plus redouté qui survient dans 8 à 12% des cas et elle est due à la sécrétion de cytokine par les cellules tumorales qui sont responsables de fréquents signes généraux à type de fièvre et d'amaigrissement [9]. Le traitement des lymphomes T/NK des fosses nasales n'est pas encore bien codifié ; il dépend principalement du stade de la maladie selon la classification d'ann arbor. Pour les stades localisés (stades I et II), une radiothérapie externe avec une dose minimale de l'ordre de 52 Gy en fractionnement classique est recommandée [10]. Elle donne une rémission complète dans 40 à 80% des cas et une survie globale à cinq ans entre 40 et 59% [11]. Pour certaines équipes, il fait généralement appel à une polychimiothérapie à base d'anthracycline suivie par une radiothérapie externe de consolidation pour les patients ayant un âge inférieur à 60 ans et le même protocole avec une polychimiothérapie sans anthracycline pour les patients de plus de 60 ans [12]. D'autres continuent à proposer de la radiothérapie seule pour les patients porteurs de lésions à stade peu avancé puisque le taux d'échecs du traitement par chimiothérapie première atteint 40%. Les patients bénéficiant d'une radiothérapie après échec de la chimiothérapie ont un meilleur pronostic [13]. Le taux de survie global, tous traitements confondus, est aux alentours de 37% [14].

## Conclusion

La localisation nasosinusienne du lymphome T/NK est rare et le diagnostic se base essentiellement sur l'immuno-histochimie. Le traitement comprend chimiothérapie et radiothérapie. Ce lymphome est de mauvais pronostic globalement, y compris sous traitement. Une meilleure prise en charge du lymphome T/NK passe par une collaboration multidisciplinaire entre médecins ORL, radio chimiothérapeutes et nutritionnistes dans le but d'améliorer le pronostic de cette pathologie.

## Conflits d’intérêts

Les auteurs ne déclarent aucun conflit d'intérêts.
